# A fluorescence spectroscopic method for rapid detection of bacterial endospores: Proof of concept

**DOI:** 10.3168/jdsc.2021-0170

**Published:** 2022-02-10

**Authors:** Nancy Awasti, Sanjeev Anand

**Affiliations:** Midwest Dairy Foods Research Center, Department of Dairy and Food Science, South Dakota State University, Brookings 57007

## Abstract

Current spore detection methods rely on culture techniques, with limitations of time, efficiency, and sensitivity. The bacterial spore coat contains calcium dipicolinic acid (CaDPA) as a major constituent, which could serve as a biomarker for bacterial endospores. We report proof of concept for a rapid and sensitive technique for the detection of bacterial endospores by using ratiometric fluorescence-based sensors. This method is based on the detection of CaDPA, which enhances the luminescence of lanthanide ions when complexed with a semiconducting polymer. A CaDPA standard curve was generated at an excitation-emission wavelength (λ) of λ_275_–λ_544_ by using a spectrophotometer. The intensity was recorded after chelating semiconducting fluorescent polyfluorene (PFO) dots with terbium (lanthanide) ions, sensitized by different volumes of CaDPA (0.1 μ*M*). The resultant standard curve showed a linear relationship (R^2^ = 0.98) in the experimental concentration range of 2.5 to 25 n*M* CaDPA, with corresponding intensity (arbitrary units) of 545 to 2,130. Endospores of the aerobic sporeformer *Bacillus licheniformis* ATCC 14580 were produced at 37°C for 15 d on brain heart infusion agar plates. The efficiency of sporulation was evaluated by spore staining and plating techniques. Total CaDPA content on spores was estimated after suspending decreasing concentrations of spores (logs 9.0 through 1.0 cfu/mL, at 1-log intervals) in HPLC-grade water (to serve as control) and skim milk samples. In HPLC-grade water, for higher spiking levels such as (mean ± SD) 9.2 ± 0.03, 8.4 ± 0.05, 7.1 ± 0.13, and 6.3 ± 0.02 logs, the corresponding mean CaDPA from the standard curve were 9.4, 7.2, 6.2, and 5.3 n*M*, respectively. For lower spiking levels of 4.2 ± 0.05, 3.1 ± 0.04, 2.0 ± 0.11, and 1.36 ± 0.09 logs, we observed mean CaDPA contents of 3.8, 3.3, 2.2, and 1.3 n*M*, respectively. For raw skim milk spiked with *B. licheniformis* ATCC 14580 spores, the mean CaDPA content on spores was approximately 2.5, 3.8, and 5.0 n*M* for spiking levels of 5.21, 6.39, and 9.47 log cfu/mL, respectively. Trials were conducted in replicates of 3 and means were compared. Trials conducted using HPLC-grade water showed a linear relationship for the CaDPA content of endospores and for endospore counts with the standard CaDPA concentration curve. For skim milk–spiked samples, we observed reduced fluorescence detection, which was 5 times lower than that of spiked samples in HPLC-grade water. The reduced fluorescence in skim milk could be due to the turbidity of the solution or to interference from proteins, amino acids, and other ions in milk. This study thus provides proof of concept for a potential application of this technique to rapidly detect bacterial endospores in the dairy and food industry. Further work is required to remove the interference of ionic components in milk to improve detection limits in milk and other dairy product matrices such as cheese, whey proteins, and reconstituted powders.

## Highlights

• Bacterial spores in milk and milk products can be detected by a rapid optical method.
• Chelated semiconducting polymer dots quantify CaDPA on spores.
• Proteins and ions in the sample may interfere with luminescence.

## Graphical Abstract

**Figure:**
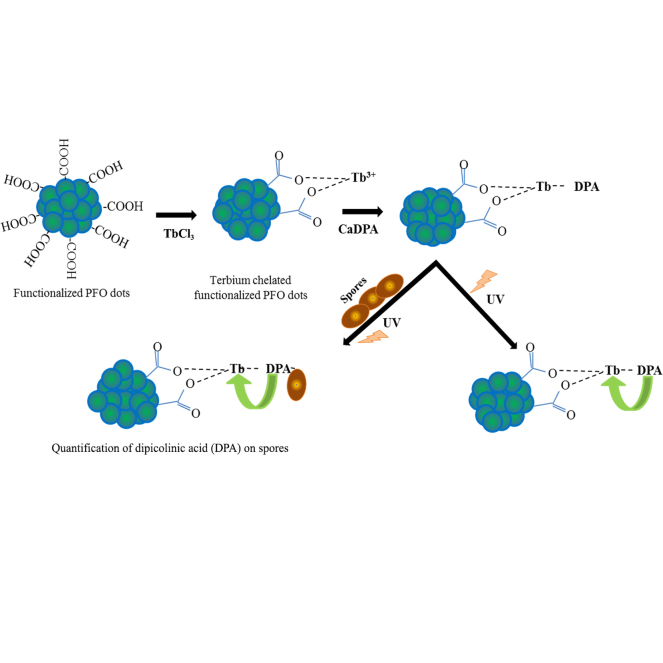
**Summary:** Bacterial endospores are resistant microbial structures that survive adverse conditions and cause product spoilage. Rapid detection of bacterial spores in dairy and food products and processing environments can thus help reduce spoilage and shelf-life issues in the final product. This study focused on evaluating a rapid, sensitive, and accurate method to quantify *Bacillus* spores in milk. The concentration of calcium dipicolinic acid (CaDPA) in *Bacillus* spores was investigated using a ratiometric fluorescence technique. CaDPA was quantified by recording the intensity (excitation-emission wavelengths 275–544 nm) of CaDPA attached to chelated semiconducting fluorescent polyfluorene (PFO). The mean CaDPA content of spiked spores detected in raw milk samples was approximately 2.5, 3.8, and 5.0 n*M* for spiked levels of 5.21, 6.39, and 9.47 log cfu/mL, respectively.

Bacterial endospores are very resistant microbial structures that survive adverse conditions and can germinate when conditions are favorable. Rapid detection of bacterial spores in dairy and food processing environments, water, and dairy and food matrices can help reduce spoilage and prevent shelf-life issues in the final product. The aerobic spore-forming *Bacillus* species is a major contaminant in the food and dairy industry (Seale et al., 2015). *Bacillus licheniformis*, being the predominant species, is frequently isolated from raw milk and responsible for significant shelf-life issues of milk and milk products (Sharma and Anand, 2002; Awasti et al., 2019). Detection and enumeration of bacterial endospore concentrations are time-consuming tasks. Therefore, timely detection of sporeformers before processing of milk is vital to identify the source of contamination and develop strategies to reduce or control *Bacillus* build-up. Several methods have been used previously, including plating techniques and molecular and optical methods. The most frequently used methods to quantify spores are microscopy and plate culture (counting) methods, which are slow and tedious, and may take up to 72 h for the results to be available. Molecular methods usually require costly reagents and require sample processing time before analysis. Thus, there remains a need for a simple and cost-effective method that can be used for the rapid identification of aerobic *Bacillus* spores in food and dairy matrices. For the past 2 decades, optical methods for the detection and enumeration of spores based on dipicolinic acid (**DPA**) have garnered attention. Some previous methods evaluated DPA as a spore marker (Bell et al., 2005) with a detection limit of 2 n*M* DPA. A study by Hindle and Hall (1999) quantified spores by monitoring spore germination and measured exuded DPA with minimum detection limit of 10^4^ *Bacillus subtilis* spores/mL.

To develop a rapid, sensitive, and accurate method to quantify spores in the food matrix, we investigated the concentration of calcium dipicolinic acid (**CaDPA**) in *Bacillus* spores using a ratiometric fluorescence technique. The strain *Bacillus licheniformis* ATCC 14580 was selected for the current study because of its prevalence and tendency for sporulation (Awasti et al., 2019). As a follow-up to a previous study (Li et al., 2013), we applied an optical method to detect bacterial spores based on detection of CaDPA, an essential biomarker and major component of bacterial spores. An enhanced emission peak with bright luminescence was observed at 544 nm upon binding of DPA with lanthanide ion (terbium, Tb^3+^), whereas the emission peak at 439 nm remained stable without addition of DPA and thus served as an internal reference (Awasti and Anand, 2018). This technique had improved detection and sensitivity when spores were spiked in HPLC-grade water, but reduced fluorescence when skim milk samples were spiked with spores. This study thus provides proof of concept for the application of this technique to rapidly detect *Bacillus* spores in the dairy and food industry, although further work is needed to reduce ionic interference in complex matrices.

Our strategy to quantify the total number of spores in ion-free water and milk was based on the detection of CaDPA content by using ratiometric fluorescence techniques, which were previously described by Li et al. (2013). Functionalized polyfluorene (**PFO**) dots were prepared using the fluorescent semiconducting polymer PFO and functional polymer poly(styrene-co-maleic anhydride) in tetrahydrofuran (THF). The detection of CaDPA using the fluorescence spectroscopic technique depends on the common absorption peak (∼275 nm) of CaDPA and the semiconducting polymer PFO. The terbium-dipicolinic acid (Tb-DPA) complex and PFO dots can be excited simultaneously at a wavelength of 275 nm without affecting the luminescence of each other. In the current study, a long-pass filter was placed in front of the detector to remove any interference from excitation at 275 nm, as reported previously (Li et al., 2013). Lanthanide ions (Ln^3+^) have a high affinity for CaDPA, and thus their binding can enable a very sensitive assay with bright luminescence. Most lanthanide-based sensors only use the change in fluorescence intensity to estimate the concentration of CaDPA, whereas the ratiometric fluorescent detection method evaluated in this study can measure the relative changes in fluorescence intensities at 2 wavelengths. The exact concentration of the analyte (in this case, CaDPA) can thus be quantitatively determined using the self-calibration curve of the ratiometric sensor. Ratiometric sensors have excellent sensitivity and selectivity, with a detection limit of 0.2 n*M*, reportedly the best among different CaDPA sensors.

Functionalized Pdots were prepared using the nanoprecipitation method described in a previous study (Li et al., 2013). The CaDPA solution was prepared by neutralizing reaction and stored at 5°C for 48 h, followed by ﬁltration and evaporation. For CaDPA sensor detection, terbium chloride (0.1 m*M* TbCl_3_) was added to an aqueous solution of prepared functionalized PFO dots to produce a reaction mixture with a Pdot concentration of about 80 p*M* and a terbium concentration of 1 μ*M*. The solution was agitated for 5 min and then luminescence sensing of the terbium-chelated PFO dots was performed by adding different volumes of CaDPA (0.1 μ*M*) to the terbium-chelated Pdot solutions. Fluorescence spectra were measured using a Synergy 2 ﬂuorescence spectrometer (BioTek Instruments Inc.). First, a standard curve was prepared by using different concentrations of CaDPA at luminescence intensity of 544 nm (Figure 1A), and then, ratios of intensities (**I**) were plotted at 544 and 439 nm (I_544_/I_439_) to generate a CaDPA calibration curve (Figure 1B). In the next step, the protocol was validated by spiking spores in HPLC-grade water. Following validation, a similar protocol was followed to determine and quantify the spores present in raw skim milk samples.

**Figure 1:**
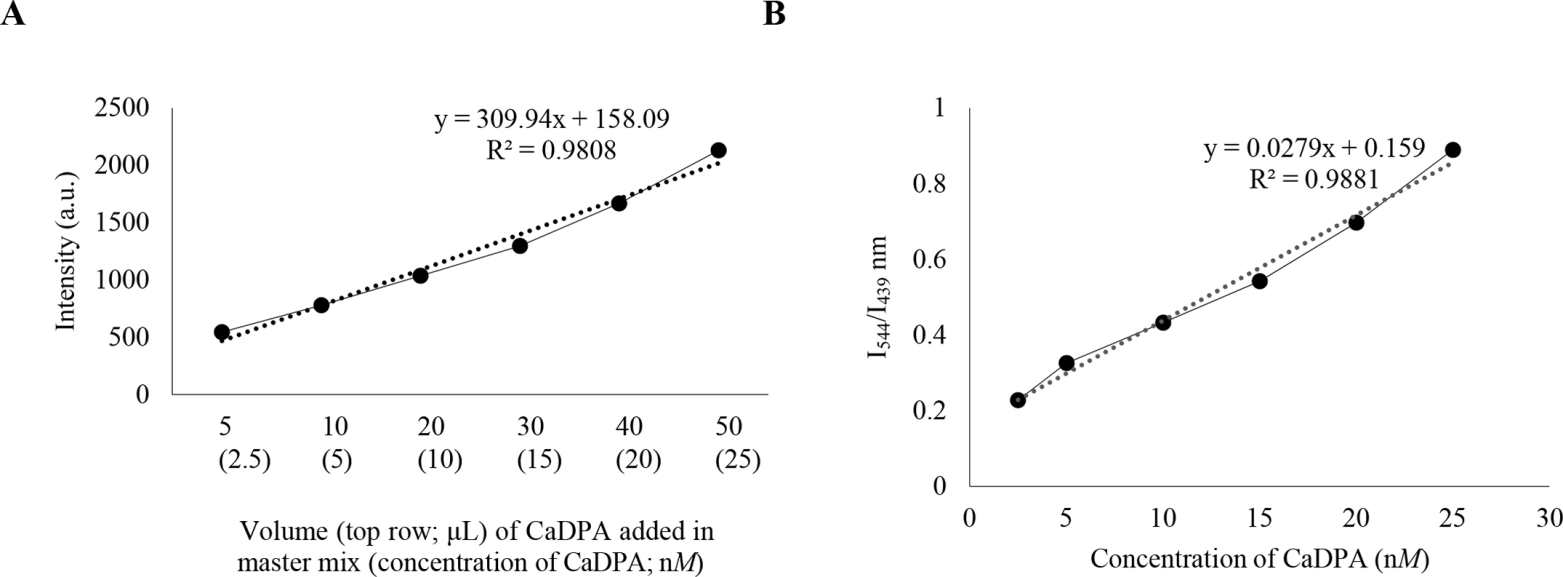
(A) Plot of change in luminescence intensity (544 nm; arbitrary units, a.u.) of the polyfluorene dot (Pdot) sensor with increasing concentrations of calcium dipicolinic acid (CaDPA). (B) Ratiometric calibration (intensity at 544 and 439 nm; I_544_/I_439_) of the Pdot sensor as a function of CaDPA concentration.

This study used *Bacillus licheniformis* ATCC 14580, purchased from the American Type Culture Collection (ATCC). The strain was propagated at 37°C in brain heart infusion agar (BHI; BD Difco), and endospores were prepared using the method described by Awasti and Anand (2018). To monitor the level of sporulation, spore staining was performed occasionally throughout the 15-d incubation period; after achieving 90% sporulation, spores were collected using centrifugation (34,000 × *g*, 15 min) followed by washing with sterile distilled water, as described by Wang et al. (2009) and Awasti (2019). Total CaDPA counts of spores spiked in HPLC-grade water and in skim raw milk samples were estimated by spiking decreasing concentrations of spores (logs 9.0 through 1.0 cfu/mL, at 1-log intervals) separately in HPLC-grade water and raw skim milk. The spiked samples were analyzed using the Synergy 2 ﬂuorescence spectrometer (BioTek Instruments Inc.). Samples were excited at a wavelength (λ) in the UV range (i.e., λ_275_), and fluorescence was read at 2 intensities: I_544_ and I_439_. The ratios of the 2 intensities were separately plotted for both sample types (Figure 1A and 1B). The mean CaDPA concentrations in spore-spiked HPLC-grade water and raw skim milk samples were quantified using the CaDPA calibration curve (Figure 1, Figure 2).

**Figure 2:**
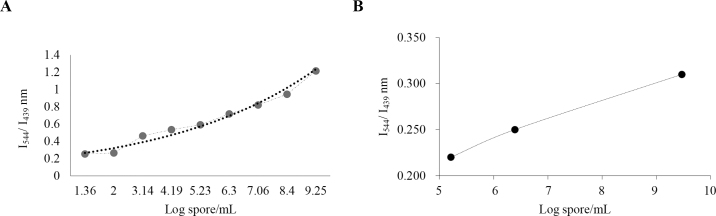
Ratiometric calibration plot (intensity at 544 and 439 nm; I_544_/I_439_) of spores spiked in (A) HPLC-grade water and (B) skim milk samples using the polyfluorene dot (Pdot) sensor as a function of calcium dipicolinic acid (CaDPA) concentration.

According to Li et al. (2013), the sensitivity of the CaDPA sensor was evaluated and their results showed a significant luminescence response of the sensor when CaDPA in aqueous solution was excited at 275 nm. The emission spectra of PFO dots and CaDPA do not interfere with each other, and their major emission peaks were at 439 and 544 nm, respectively. Our results validated those of the previous study by plotting the luminescence intensity with increasing CaDPA concentration. Our results agreed with those of Li et al. (2013) in terms of excitation and emission of CaDPA sensors; CaDPA was detected and plotted at excitation-emission (λ_275_-λ_544_) wavelengths using the Synergy 2 fluorescence spectrophotometer (Figure 1A). A linear relationship (R^2^ = 0.98) was observed for our experimental CaDPA concentration range of 2.5 to 25 n*M* with corresponding intensity (arbitrary units, a.u.) of 545 to 2,130.

Figure 1B explains the ratiometric calibration plot (I_544_/I_439_) of the Pdot sensor as a function of CaDPA concentration. The ratios of 2 emission intensities were plotted where the highest emission peaks of Tb^3+^ were excited after chelating it between DPA and functionalized PFO dots. In terms of sensor sensitivity, our results agree with the previous report, and the limit of detection observed was approximately 0.2 n*M*. The results shown in Figure 1B allowed us to create a link between CaDPA concentration and ratiometric intensity, which was further used to calibrate the total CaDPA content of spores spiked in HPLC-grade water and raw skim milk samples. After exciting the reaction mixture of spores spiked in HPLC-grade water at 275 nm, luminescence was observed at 2 intensities: I_544_ and I_439_. The ratios of these 2 intensities were plotted against spiked log spore counts (log cfu/mL), as shown in Figure 1A. This graph was compared with the CaDPA calibration curve (Figure 1B) to quantify the total concentration of CaDPA on spores. For higher spiking levels such as 9.2 ± 0.03, 8.4 ± 0.05, 7.1 ± 0.13, and 6.3 ± 0.02 logs, the corresponding mean CaDPA values determined from the standard curve were 9.4, 7.2, 6.2, and 5.3 n*M*, respectively. For the lower levels of 4.2 ± 0.05, 3.1 ± 0.04, 2.0 ± 0.11, and 1.36 ± 0.09 logs, we determined mean CaDPA concentrations of 3.8, 3.3, 2.2, and 1.3 n*M*, respectively. The mean CaDPA concentration on spores ranged from approximately 30 to 3 n*M*. Trials conducted using HPLC-grade water indicated a linear relationship of CaDPA content of endospores with the endospore counts and the standard curve of CaDPA concentration.

For raw skim milk spiked with *B. licheniformis* ATCC 14580 spores, the mean CaDPA concentrations detected on spores were approximately 2.5, 3.8, and 5.0 n*M* for spiking levels of 5.21, 6.39, and 9.47 log cfu/mL, respectively (Figure 2B). Under our test conditions, we could not detect intensities below the spiking level of 5.21 log cfu/mL. The fluorescence detection was approximately 5 times lower for spiked samples in skim milk compared with those in HPLC-grade water. The reduced fluorescence in raw milk may be due to the turbidity of the solution or interference from proteins, amino acids, and other ions present in milk. To improve the ability of the sensor, additional strategies are needed to remove interfering components such as proteins and other ions. This study supports the potential application of this technique to rapidly detect bacterial endospores in the dairy and food industry. Further refinements are needed to remove the interference of ionic components in milk and improve the efficiency of the sensor to rapidly detect spores.
